# Research Progress on Diseases and Pests of Chrysanthemum (2015–2025)

**DOI:** 10.3390/ijms26199767

**Published:** 2025-10-07

**Authors:** Yuan Chen, Lihui Han, Tengqing Ye, Chengjian Xie

**Affiliations:** 1 Institute of Vegetables and Flowers, Chongqing Academy of Agricultural Sciences, Chongqing 401329, China; eddygemini@163.com; 2 The College of Life Science, Chongqing Normal University, Chongqing 401331, China; hanlihui999@163.com (L.H.); 15223536067@163.com (T.Y.)

**Keywords:** chrysanthemum, fungal diseases, bacterial pathogens, viruses, insect pests, resistance breeding, molecular diagnostics, integrated pest management

## Abstract

*Chrysanthemum morifolium* Ramat. is a major ornamental crop that suffers from diverse fungal, bacterial, viral, and insect pests, causing significant yield and quality losses. Between 2015 and 2025, rapid progress in molecular biology, genomics, and ecological regulation has advanced both fundamental research and applied control strategies. Multi-locus sequencing, multiplex PCR, and next-generation sequencing refined the identification of fungal and bacterial pathogens, while functional studies of WRKY, MYB, and NAC transcription factors revealed key resistance modules. Hormone-mediated signaling pathways, particularly those of salicylic acid, jasmonic acid, and abscisic acid, were shown to play central roles in host defense. Despite these advances, durable genetic resistance against bacterial pathogens and broad-spectrum defense against viruses remains limited. Novel technologies, including virus-free propagation, RNA interference, and spray-induced gene silencing, have shown promising outcomes. For insect pests, studies clarified the damage and virus-vectoring roles of aphids and thrips, and resistance traits linked to trichomes, terpenoids, and lignin have been identified. Biocontrol agents such as *Trichoderma* spp., *Bacillus* spp., predatory mites, and entomopathogenic fungi have also demonstrated efficacy. Future efforts should integrate molecular breeding, genome editing, RNA-based tools, and microbiome management to achieve sustainable chrysanthemum protection.

## 1. Introduction

Chrysanthemum (*Chrysanthemum morifolium* Ramat.) is one of the most important ornamental flowers worldwide. Over the decade 2015–2025, chrysanthemum production has faced numerous challenges from pathogens and pests. This review summarizes research progress from 2015 to 2025, focusing on fungal diseases, bacterial diseases, viral and viroid diseases, and insect pests (Table 1). For each category, we highlight advances in pathogen identification and classification, host resistance mechanisms, resistance breeding, and innovative control strategies including biocontrol, RNA interference, and novel chemical agents.

## 2. Fungal Diseases

### 2.1. Major Fungal Diseases of Chrysanthemum and Their Diagnostic Identification

Chrysanthemums are highly susceptible to diverse fungal pathogens that cause leaf spot, wilt, rust, blight, and rot, leading to significant yield and quality losses [1]. Advances in molecular technology have improved the precision of fungal pathogen identification. For example, a severe outbreak of chrysanthemum leaf spot in Zhejiang, China in 2022 was confirmed to be caused by *Nigrospora oryzae*, a pathogen previously known mainly on rice. This was the first global report of *N. oryzae* infecting chrysanthemum, confirmed by multi-locus DNA sequencing [2]. Similarly, *F. oxysporum* f. sp. *chrysanthemi*, historically regarded as the causal agent of chrysanthemum wilt, has been reclassified. Cases in India between 2019 and 2020 revealed that the actual pathogen was *F. incarnatum*, a member of the incarnatum-equiseti species complex, confirmed through multi-gene phylogenetic analysis and Koch’s postulates [3]. Such findings highlight cryptic speciation within Fusarium and refine our understanding of wilt etiology. A 2023 survey in Lam Dong, the main chrysanthemum-producing region of Vietnam, showed that the wilt pathogens are dominated by *F. oxysporum* (50%) and *F. falciforme* (48%). The two species display a “complementary” field distribution: the former preferentially infects cuttings, whereas the latter favors tissue-cultured plantlets, indicating that control measures should be tailored to the propagation material used [4]. Some foliar diseases such as chrysanthemum *Alternaria alternata* (leaf blight) [5], *Botrytis cinerea* [6], *Nigrospora sphaerica* (leaf spot) [7], *Golovinomyces* sp. (species indeterminate, Powdery mildew) [8] and anthracnose caused by *Colletotrichum siamense* have been reported in Asia [9]. Further studies have shown that the host range of *G. cichoracearum* includes chrysanthemum [10]. Moreover, in chrysanthemum production, soil-borne diseases such as *Sclerotinia sclerotiorum* and *Verticillium dahliae* are also notoriously difficult to control [11,12]. Southern blight of *C. morifolium* in Hubei (30–50% incidence) was caused by *S. rolfsii*, confirmed by morphology, sequencing, and pathogenicity; this is the first report in China [13]. While microscopy and culturing remain valuable, they are increasingly complemented or replaced by PCR-based diagnostics. For instance, in situ hybridization (ISH) revealed that *Puccinia horiana* (white rust) could establish systemic infections in asymptomatic leaves, with hyphae and haustoria distributed in distal leaf tissues [14]. A multiplex real-time PCR assay targeting the rDNA ITS region was established to differentiate *P. horiana* (white rust, regulated) from *P. chrysanthemi* (brown rust, less harmful). The method detects as little as 1 pg DNA, identifies pathogens in fresh and herbarium samples with 99% success, and provides a practical tool for accurate *P. horiana* quarantine diagnosis [15].

### 2.2. Physiological Defense Mechanisms and Resistance Research of Chrysanthemum Against Fungal Diseases

*A. alternata* is one of the most important necrotrophic fungi causing black spot disease in chrysanthemum, and is also the most extensively studied fungal pathogen. Host resistance to *A. alternata* is closely linked to anatomical traits. During an *A. alternata* epidemic in Egypt in 2018, resistant cultivars such as ‘Podolsk Purple’ showed thicker cuticles and tissues compared to susceptible ones, contributing to resistance [16]. RNA-seq and weighted gene co-expression network analysis (WGCNA) revealed stage-specific defenses of chrysanthemum against *A. alternata*: *Abscisic Acid*/*Salicylic Acid*/*Enhanced Disease Susceptibility 1* (ABA/SA/EDS1) signaling in early infection, Ethylene (ET) and Ca^2+^ pathways during lesion formation, and late-stage induction of MATE genes likely exporting pathogen toxins. qPCR and transgenic validation of *hub* genes provide molecular targets for *A. alternata* resistance breeding [17]. *A. alternata* effector Alta1 induces cell death and defense by activating Jasmonic Acid (JA) signaling in chrysanthemum. It interacts with circadian-related CmWD40, whose overexpression promotes JA accumulation and MYC2 transcription, enhancing resistance; silencing of *CmWD40* reduces resistance. The Alta1-CmWD40-JA-MYC2 module is thus central to defense against *A. alternata* [18]. Several potential resistance resources have been identified. For example, the NAC transcription factor CmNAC083, localized in the nucleus, was strongly induced by *A. alternata*. The overexpression of *CmNAC083* enhanced resistance to black spot disease, while silencing caused susceptibility. CmNAC083 confers defense by activating JA and ROS pathways [19]. In chrysanthemum, CmMLO17 is induced by *A. alternata* infection. Silencing CmMLO17 reduces susceptibility via enhanced ABA and Ca^2+^ signaling. The interactor of CmMLO17, CmKIC, was confirmed to localize on the plasma membrane. Specifically, the overexpression of CmKIC increased the susceptibility of chrysanthemum to *A. alternata*, whereas its silencing led to a decrease in such susceptibility. Together, the CmMLO17-CmKIC module promotes pathogen growth and represents a key susceptibility factor [20]. Hormone signaling pathways were also shown to play crucial roles. Pretreatment with methyl jasmonate (MeJA) reduced chrysanthemum susceptibility to *A. alternata*. Metabolome and transcriptome analyses showed that JA signaling enhanced resistance through cell wall modification, Ca^2+^/ROS regulation, MAPK pathways and hormonal pathways, and antifungal metabolite accumulation. Validated by qPCR and transgenic assays, this finding indicates that MeJA is a promising eco-friendly strategy to prime broad-spectrum defense against black spot disease [21]. Grafting chrysanthemums onto *Artemisia vulgaris* rootstocks increased resistance to *A. alternata* by raising JA levels, which promoted trichome density, terpenoid accumulation, and CmJAZ1-like degradation. CmJAZ1-like protein acted as a negative regulator, with silencing enhancing and overexpression reducing resistance [22]. In resistant chrysanthemum cultivar ‘Huaiju 2’, *Alternaria* infection elevated SA/JA levels and defense enzyme activities. RNA-Seq showed differentially expressed genes (DEGs) in R proteins, ROS, Ca^2+^, MAPK, and JA signaling, with strong activation of the SA pathway. Overexpression of CmNPR1 further enhanced resistance, confirming SA signaling as central to black spot defense [23] (Table 2).

RNA-seq of chrysanthemum ‘Jinba’ under *F. oxysporum* infection identified 7985 DEGs enriched in MAPK, secondary metabolism, and sugar pathways. Early defense involved phenolic compound biosynthesis, proline accumulation, sugar reduction, and a candidate WRKY transcription factor regulating resistance [24]. Functional analysis identified CmWRKY6-1 as a nuclear transcriptional repressor that negatively regulates resistance by modulating ROS and SA pathways. CmWRKY6-1 directly suppresses CmWRKY15-like, and silencing CmWRKY15-like reduced resistance, establishing the CmWRKY6-1-CmWRKY15-like cascade as a key regulator of Fusarium wilt immunity in chrysanthemum ‘Jinba’ [25]. *F. oxysporum* root infection significantly induced terpene production in chrysanthemum roots and leaves, with sesquiterpenes dominant and monoterpenes leaf-specific. Transcriptome analysis identified 8 TPS genes whose expression matched terpene accumulation; biochemical assays confirmed functions of Cm-j-TPS1/2/7. Infection also elevated SA levels in roots and leaves, linking SA signaling to defense [26]. In chrysanthemum ‘Huangju’, *F. oxysporum* induced stage-specific responses: galactose metabolism across all phases, with auxin/ABA/ET early, auxin/SA and TFs (e.g., CmWRKY48) in the middle, and galactose metabolism dominating late. Silencing *CmWRKY48* enhanced resistance, identifying it as a negative regulator and potential breeding target [27]. In contrast, overexpression of the CmWRKY8-1-VP64 fusion protein downregulated SA biosynthetic genes PAL and EDS1 by more than 50%, rendering plants more susceptible to *F. oxysporum*, further underscoring the central role of the WRKY–SA module in wilt resistance [28] (Table 2).

White rust (*P. horiana*) severely limits chrysanthemum production, and breeding resistant cultivars is crucial. Using genome-wide association study (GWAS) on an F1 biparental population (‘Southern Pegasus’ × susceptible line), 21 SNPs were associated with resistance, forming one linkage group. A DNA marker 2.2 cM from the resistance locus was identified and validated, representing the first effective marker linked to *P. horiana* resistance in chrysanthemum [29]. In Indonesia, commercial production of *C. morifolium* var. Mustika Kaniya is constrained by *P. horiana*. Histological comparison showed pustules on abaxial leaf epidermis and significant reductions in leaf (epidermis, mesophyll, vascular tissues) and stem (epidermis, cortex, bundle sheath, pith) structures, indicating major tissue deformation caused by infection [30]. The transcription factor CmTGA1 was found to regulate resistance by activating CmRbohD, which promotes ROS generation, antioxidant enzyme activity, and lignin biosynthesis. Overexpression of CmTGA1 enhanced, while knockout reduced resistance. The CmTGA1-CmRbohD cascade thus underpins ROS-mediated defense and offers a strategy for breeding *P. horiana*-resistant cultivars [31]. The transcription factor CmWRKY15-1 positively regulated resistance by enhancing defense enzyme activity and reducing H_2_O_2_. RNA-seq and functional assays showed that CmWRKY15-1 binds the promoter of CmNPR1, activating the SA pathway and downstream PR genes. The CmWRKY15-1-CmNPR1 module thus plays a central role in *P. horiana* resistance [32]. CmCC-NB-ARC was identified by NBS-domain search and showed eight nonsynonymous mutations between resistant and susceptible cultivars. Expression patterns differed between resistant ‘C029’ and susceptible ‘LZ08-61’. Overexpression of CmCC-NB-ARC in susceptible ‘Jinba’ enhanced resistance, confirming its role in *P. horiana* defense and providing a valuable target for resistance breeding [33] (Table 2).

In chrysanthemum, both *B. cinerea* infection and mechanical injury induce emission of overlapping VOCs, including green leaf volatiles, terpenes, and phenylpropanoid derivatives, reflecting a shared defense response [34]. Phenylalanine (Phe) pre-treatment further enhances resistance to *B. cinerea* by promoting antifungal metabolites (e.g., phenylacetaldehyde, eugenol), cell wall precursors, and stabilizing host metabolism, while reducing ROS and ethylene and activating Ca^2+^/hormonal signaling. Collectively, VOC emission and Phe priming synergistically reinforce broad-spectrum defense in chrysanthemum [6] (Table 2).

**Table 2:** Molecular and Physiological Responses, Breeding Strategies to major fungal pathogens.

Pathogen	Factor/Gene/Approach	Mechanism and Effect	References
*A. alternata* (black spot)	Anatomical traits	Resistant cultivars had thicker cuticle and tissues	[16]
	ABA/SA/EDS1, ET, Ca^2+^, MATE genes	Stage-specific signaling (early ABA/SA, lesion ET/Ca^2+^, late detoxification); hub genes identified for breeding	[17]
	Alta1–CmWD40–JA–MYC2	Activates JA signaling; overexpression enhanced resistance, silencing reduced resistance	[18]
	CmNAC083	NAC transcription factor activating JA and ROS pathways; overexpression enhanced resistance, silencing reduced resistance	[19]
	CmWRKY6-1—CmWRKY15-like	Negative regulator suppressing ROS and SA signaling	[25]
	CmMLO17–CmKIC	Promotes susceptibility via ABA/Ca^2+^ signaling; silencing reduced susceptibility	[20]
	MeJA priming	Activates JA defense pathways; reduced susceptibility	[21]
	Grafting with *A. vulgaris*	Increased JA, trichomes, and terpenoids; CmJAZ1-like identified as negative regulator	[22]
	CmNPR1	Activates SA pathway; overexpression enhanced resistance in ‘Huaiju 2’	[23]
*F. oxysporum* (wilt)	DEGs (MAPK, phenolics, sugars)	Early phenolic biosynthesis, sugar reduction, transcription factor regulation of resistance	[24]
	CmWRKY6-1—CmWRKY15-like	Negative SA/ROS regulator; suppressed wilt resistance	[25]
	TPS genes	Infection induced sesquiterpenes and monoterpenes; SA signaling involved	[26]
	CmWRKY48	Negative regulator via SA/auxin/ABA pathways; silencing enhanced resistance	[27]
	CmWRKY8-1–VP64	Downregulated SA biosynthetic genes; overexpression reduced resistance	[28]
*P. horiana* (white rust)	GWAS/QTL	21 SNPs identified; DNA marker linked to resistance breeding	[29]
	Histological changes	Severe tissue deformation in susceptible cultivars	[30]
	CmTGA1–CmRbohD	ROS and lignin cascade; overexpression enhanced resistance, knockout reduced resistance	[31]
	CmWRKY15-1–CmNPR1	Activated SA pathway and PR genes; positive regulator of resistance	[32]
	CmCC-NB-ARC	NBS-LRR variants; overexpression enhanced resistance	[33]
*Botrytis cinerea* (gray mold)	VOC emission	Shared antifungal signals with wounding; induced broad-spectrum defense	[34]
	Phenylalanine priming	Promoted antifungal metabolites, stabilized metabolism, reduced ROS and ethylene	[6]

### 2.3. Biocontrol Strategies Against Fungal Diseases in Chrysanthemum

Biocontrol and cultural strategies for chrysanthemum diseases have progressed on multiple fronts [35]. *Trichoderma harzianum* promotes rooting of chrysanthemum cuttings and reshapes both endophytic and rhizosphere microbiomes, changes that are associated with improved growth and pathogen suppression [36]. Biochar (BC) is a carbon-rich, porous material derived from the pyrolysis of biomass (e.g., crop residues, wood chips, or manure) under limited oxygen conditions. As a soil amendment, biochar improves the physicochemical environment and provides microhabitats for beneficial microorganisms, while *B. subtilis* (BM) acts as a potent microbial antagonist. Their combined application (BM_BC) in chrysanthemum soils effectively suppressed *F. oxysporum*, enhanced root activity and plant biomass, and promoted greater microbial diversity, demonstrating strong synergistic effects for the sustainable management of Fusarium wilt [37]. In chrysanthemum monoculture soils, *B. subtilis* biofungicide enriched beneficial microbes and reduced *F. oxysporum* by 79%, sustainably suppressing Fusarium wilt, while fumigant dazomet only gave short-term control with pathogen rebound [38]. Against emerging *N. oryzae* leaf spot, *B. siamensis* D65 isolated from the chrysanthemum phyllosphere showed the strongest in vitro antagonism among tested strains [2]. For chrysanthemum white rust, *Cladosporium cladosporioides* and *C. pseudocladosporioides* parasitize *P. horiana* teliospores, exhibit β-1,3-glucanase activity, and reduced disease index in greenhouse sprays, indicating an enzymatic-degradation plus competition mode of action [39].

Natural compounds are also promising: *Artemisia* spp. showed strong aphid repellence and antifungal activity compared with chrysanthemum. Among them, leaf and stem extracts of *A. maximowicziana* had the highest inhibition of *A. alternata*, *C. siamense*, and *Phoma* sp., while root extracts displayed only weak activity against *F. solani*. GC-MS and OPLS-DA identified terpenoids as major volatiles, with (-)-thujol the key antimicrobial component. *A. maximowicziana* thus represents a valuable parent for breeding resistant chrysanthemums [40]. Intercropping chrysanthemum with ginger suppressed Fusarium wilt and boosted biomass by enriching *Burkholderia* spp. via ginger root exudates, which enhanced rhizosphere colonization and biofilm formation, highlighting a microbiota-mediated mechanism of disease suppression [41]. In field trials with *C. morifolium* ‘Hangbaiju’, rice-straw biochar improved soil pH, organic carbon, potassium, and phosphorus contents but reduced available nitrogen. At a 10% application rate, biochar optimized soil microbial communities by increasing bacterial and actinomycete populations while reducing fungi, with *F. oxysporum* decreased by 42.4–54.4%. This shift contributed to a 23.9% increase in yield and a significant enhancement of flavonoid content in chrysanthemum flowers. Thus, 10% biochar was most effective in improving soil quality, suppressing pathogens, and enhancing production [42].

## 3. Bacterial Diseases

### 3.1. Major Bacterial Diseases of Chrysanthemum and Their Diagnostic Identification

Compared with fungi, relatively few bacterial species infect chrysanthemum, yet their outbreaks can result in severe economic losses. Reported pathogens include *Ralstonia solanacearum* (bacterial wilt) [43], *Pseudomonas cichorii* and *P. putida* (leaf spot/blight) [44,45], *Dickeya chrysanthemi* (stem rot) [46], *Agrobacterium rubi* and *A. tumefaciens* (crown gall) [47]. Chrysanthemum fasciation, caused by *Rhodococcus fascians*, produces hormone-like compounds that disrupt development, leading to malformed buds, shortened internodes, and stunted growth. The pathogen spreads via infected cuttings and is difficult to eradicate, often requiring plant removal and soil disinfection [48]. Taxonomic revisions have clarified pathogen identities; for instance, soft rot once attributed to *Erwinia chrysanthemi* is now classified under *D. chrysanthemi* [46]. *R. solanacearum* remains highly destructive in subtropical regions and greenhouse environments, persisting in soil and water and disseminating through contaminated cuttings and tools, underscoring the importance of early detection [43]. *P. cichorii* causes dark, water-soaked lesions that later turn necrotic. Phylogenetic analyses have revealed considerable genetic diversity and novel lineages of *P. cichorii* linked to outbreaks in ornamentals, including chrysanthemum in Florida [49].

### 3.2. Management Strategies for Bacterial Diseases of Chrysanthemum

Unlike fungal pathogens, strong genetic resistance sources against bacterial diseases in chrysanthemum are largely lacking. Nevertheless, several host responses and management strategies have been documented. For instance, *P. cichorii* secretes AvrE1 effector proteins that enhance virulence; mutants deficient in AvrE1 cause significantly milder symptoms, highlighting potential molecular targets for resistance breeding [50]. *P. putida* was identified as a causal agent of bact. blight on *Chrysanthemum* in Karnataka, India. Bactinash and Anucin were effective bactericides, while bio-agents (*T. viride*, *P. fluorescens*) also suppressed the pathogen. This was the first report of *P. putida* infecting chrysanthemum in India [45]. Other plant-derived genes may also serve as resistance candidates. For example, the peanut NBS-LRR gene *AhRRS5*, localized in the nucleus, was induced by *R. solanacearum*, hormones (SA, ABA, MeJA, ET), and abiotic stresses. Its overexpression triggered hypersensitive response in *Nicotiana* and enhanced tobacco resistance, accompanied by activation of SA/JA/ET signaling and defense genes such as *NPR1*. Thus, *AhRRS5* mediates multi-pathway defense against bacterial wilt [51].

*R. solanacearum* remains a devastating pathogen with a broad host range. *R. solanacearum* infection reduced soil ammonium and plant nitrogen, disturbed rhizosphere and endophyte microbes, decreased *Rhodanobacter*, and enhanced denitrification. Balanced nitrogen management and microbiome regulation may help control bacterial wilt [52]. A combined strategy of ammonium bicarbonate fumigation with organic amendments has been shown to markedly reduce disease severity by reshaping soil microbial communities, with shifts in the rhizosphere bacterial composition—particularly the enrichment of *Rhodanobacter*, *Terrimonas*, and *Chitinophaga*—identified as key suppressive factors [53]. Companion planting has also demonstrated promise; intercropping tomato with basil or cilantro suppressed *R. solanacearum* incidence while enriching beneficial taxa such as *Pseudomonas* and *Aquabacterium* in the rhizosphere [54]. Numerous biocontrol agents, including *Bacillus subtilis* SYST2, *Paenibacillus*, *Pseudomonas*, and *Serratia*, as well as fungi such as *Trichoderma* and the oomycete *Pythium oligandrum*, suppress *R. solanacearum* through mechanisms of antibiosis, competition, and induction of host resistance [55,56,57]. Three lytic Podoviridae phages from Spanish rivers effectively lysed *R. solanacearum*, persisted in water for months, and reduced wilt incidence via irrigation, showing strong potential as sustainable biocontrol agents [58]. In addition, the incorporation of organic amendments into soil-such as green manure, essential oils (e.g., lemongrass oil), livestock manure, and small organic molecules including lysine, riboflavin, γ-aminobutyric acid, and methyl gallate-can control the pathogen either by altering soil microbial activity or through direct inhibition of *R. solanacearum* [59,60]. Alkyl gallates also were tested against *R. solanacearum*, with methyl gallate showing the strongest activity, decreasing with longer ester chains. Methyl 2,3-dihydroxybenzoate exhibited similar or stronger effects, while the plant metabolite geraniin showed moderate activity [60].

## 4. Viral and Viroid Diseases

### 4.1. Major Viral and Viroid Diseases of Chrysanthemum and Their Diagnostic Identification

Viruses and viroids have long posed serious challenges to chrysanthemum cultivation, causing symptoms such as mosaic, chlorotic mottle, stunting, and flower breaking. Because propagation primarily relies on cuttings and tissue culture, infected materials readily facilitate the spread of these pathogens [61]. To date, more than 20 viruses and at least two viroids have been reported to infect chrysanthemum [62]. The major viruses include chrysanthemum virus B (CVB), chrysanthemum virus R (CVR), cucumber mosaic virus (CMV), tomato spotted wilt virus (TSWV), impatiens necrotic spot virus (INSV), potato virus Y (PVY), chrysanthemum stem necrosis virus (CSNV), and tobacco mosaic virus (TMV) [63,64]. The viroids identified are chrysanthemum stunt viroid (CSVd) and chrysanthemum chlorotic mottle viroid (CChMVd) [65]. In the past decade, several novel viruses have been characterized in chrysanthemum. For instance, chrysanthemum virus D (ChVD) was identified as a new *Polerovirus*, sharing less than 75% sequence similarity with known members [66]. Chrysanthemum yellow dwarf-associated virus (CYDaV) was described as a novel *Cytorhabdovirus* [67]. Chrysanthemum mosaic-associated virus (ChMaV) was assigned to the genus *Emaravirus*, closely related to pear chlorotic leaf spot-associated virus [68]. More recently, chrysanthemum sadwavirus (ChSV) was identified as a new *Sadwavirus* with a bipartite RNA genome, sharing only 53% sequence similarity with *Lettuce secovirus 1* [69].

Recent advances have greatly enhanced both the detection and the mechanistic understanding of chrysanthemum viruses and viroids. Multiplex RT-PCR and RT-qPCR kits are now available for routine diagnosis of CVB, CMV, TAV, INSV, TSWV, CSVd, and CChMVd [70]. Additional multiplex assays have been developed for TSWV, DMV, and CSVd [71], and duplex RT-PCR methods for TSWV and CSVd offer high sensitivity and specificity [72]. RT-LAMP systems provide a rapid alternative, completing detection within 12–30 min with sensitivity equal to or greater than RT-PCR, and have been applied to CSNV, TMV, and CSVd [61,73,74]. Moreover, NGS-based metatranscriptomics and small RNA sequencing enable simultaneous detection of multiple viruses and reveal complex co-infections [62].

### 4.2. Pathogenic Mechanisms and Resistance Research on Major Viral and Viroid Diseases of Chrysanthemum

Several viral pathogenic mechanisms have been documented. CVB encodes the multifunctional p12 protein, which functions as a nuclear transcriptional activator and a cytoplasmic RNA silencing suppressor. p12 alters sRNA profiles by reducing overall accumulation, shifting siRNA size distribution and strand ratios, decreasing 5′-U siRNAs, and downregulating several miRNAs [75]. Similarly, a 27-nt insertion in TAV 2b enhances its RISC affinity, strengthening systemic infection in Iranian isolates [76]. Viroids also exploit RNA silencing; for example, CChMVd-derived sRNAs target chloroplast genes to induce chlorosis, with evidence suggesting seed transmission [77,78]. Moreover, tospovirus NSm proteins are central to intercellular movement and systemic infection, but also serve as elicitors of *Sw-5b*–mediated resistance. The tomato R gene *Sw-5b* encodes a CC-NBS-LRR protein that recognizes the NSm proteins of TSWV, CSNV, and TCSV, thereby triggering a hypersensitive response. Point mutations at NSm residues Cys118 or Thr120 abolish recognition, while the BeNMV NSm is naturally unrecognized, underscoring the site-specific nature of R gene perception [79,80]. Mechanistic studies have revealed extensive host transcriptional reprogramming in response to viral infection. CMV, TSWV, and PVX each induced about 100 differentially expressed genes, with conserved responses observed in ethylene signaling and DNA metabolism [81]. NGS-based transcriptomics of a Chinese isolate (CVB-CN) identified 4934 SDEGs, mainly enriched in ethylene signaling, phenylpropanoid/flavonoid biosynthesis, and ribosome metabolism. Ethylene pathway activation was confirmed as critical for resistance, as silencing this pathway in *Nicotiana benthamiana* increased susceptibility to CVB [82]. In chrysanthemum ‘Hangju’, virus-free plants reinfected with CVB showed improved yield and medicinal quality. Transcriptomics (6223 DEGs) indicated reduced metabolic stress and activation of SA/PAL-mediated defense, suggesting virus-free technology reshapes host responses [83].

Although some older cultivars show tolerance to CSVd, displaying only mild stunting despite infection, the absence of natural resistance genes makes breeding for virus resistance particularly difficult [65]. To date, only a limited number of resistance genes have been reported. CmNF-YB8 negatively regulates defense by repressing CmCIPK6 and CmSHN3, and its silencing enhances cuticle deposition and resistance to CVB and TSWV [84]. Consequently, current management strategies focus on the production of virus-free stock through meristem tip culture, thermotherapy, and shoot tip cryotherapy, which often achieve complete pathogen elimination [85,86]. Low-temperature treatment has been shown to displace CSVd from shoot apical meristems and confine it to vascular tissues, thereby suppressing replication and movement and providing a mechanistic basis for eradication [87,88]. The medicinal chrysanthemum cultivar ‘Huaihuang’ suffers yield losses from tomato aspermy virus (TAV). A combined shoot apex culture and heat treatment produced up to 80% TAV-free plants, resulting in an elite line with enhanced growth, flower yield, pigment accumulation, enzyme activity, and secondary metabolite levels [89].

Transgenic resistance via RNA interference has also been tested. For instance, Transgenic chrysanthemums expressing CVB coat protein **or** RNAi constructs showed variable resistance, with some double-sense lines fully resistant and RNAi lines markedly reducing infection, demonstrating the potential of genetic engineering for CVB resistance [90]. Beyond genetic and molecular strategies, novel approaches such as dsRNA spray-induced gene silencing [91], which reduced virus titers and mitigated symptoms, and the use of UV-absorbing greenhouse covers to suppress vector populations like thrips and aphids, further broaden the arsenal of tools available for virus management in chrysanthemum [92]. In South Korea, TSWV was detected in 70.77% of chrysanthemums and 72.96% of thrips (*Frankliniella occidentalis*)**.** A combined treatment with *Stratiolaelaps scimitus* and four essential oils reduced TSWV incidence, offering an eco-friendly control strategy [93]. Similarly, *B. velezensis* VB7 exhibited broad antiviral and antifungal activity, producing lipopeptides and VOCs that inhibited TSWV and CVB, while field application enhanced plant growth and floral yield [94]. In addition, prohydrojasmon (PDJ), a synthetic jasmonate derivative, effectively reduced thrips feeding damage, larval reproduction, and TSWV transmission without causing phytotoxicity at optimal rates. These findings highlight PDJ as a promising eco-friendly strategy for managing thrips and orthotospoviruses in chrysanthemum [95].

## 5. Insect Pests

### 5.1. Major Insect Pests of Chrysanthemum

Chrysanthemum production is challenged by aphids (*Macrosiphoniella sanborni*, *Myzus persicae*, *Aphis gossypii, A. annua*) [96], thrips (*F. occidentalis*) [97], whiteflies (*Trialeurodes vaporariorum*, *Bemisia tabaci*) [98], leafminers (*Liriomyza* spp.) [99], spider mites (*Tetranychus urticae*) [100], and occasional lepidopteran pests (*Helicoverpa armigera*) [101]. *A. gossypii* is a major chrysanthemum pest, infesting leaves and buds at all stages. Heavy infestations cause malformation, reduced vigor, and death, while honeydew-induced sooty mold lowers photosynthesis and market value. It also transmits Cucumber mosaic virus and Chrysanthemum stunt viroid, intensifying losses [102]. *M. sanborni* feeding impairs phloem sugar unloading in chrysanthemum, reducing soluble sugars by 19%, and also transmits Chrysanthemum virus B (CVB) non-persistently, with mottling incidence reaching 63% at 7 d [103]. Thrips feeding results in silvering of leaves and floral malformations, while also transmitting viruses [104]. Surveys in Bangladesh identified thrips as the most damaging pest, followed by aphids [105].

### 5.2. Physiological Defense Mechanisms and Resistance Research of Chrysanthemum Against Insect Pests

Breeding efforts have revealed natural variation in resistance. Resistant traits include tougher leaves, higher trichome density, or elevated secondary metabolites such as terpenoids and polyacetylenes [106]. Aphid feeding altered VOC profiles in *A. annua* and chrysanthemum; bioassays showed species-specific preferences, with elevated (E)-β-farnesene and artemisia ketone implicated in resistance [107]. Transcription factors play pivotal roles in regulating insect resistance in chrysanthemum. CmWRKY48 and CmMYB15 were both induced by aphid infestation. Overexpression of CmWRKY48 suppressed aphid population growth, while CmMYB15 bound AC elements of lignin biosynthesis gene promoters, promoted lignin accumulation and related gene expression, thereby enhancing aphid resistance [108,109]. Similarly, the R2R3-MYB factor *CmMYB15* and *CmMYB19* was aphid-inducible, and its overexpression restricted aphid multiplication through the upregulation of lignin biosynthesis genes and increased lignin deposition [108,110]. In contrast, *CmWRKY53* was also induced by aphids but acted as a negative regulator: overexpression increased plant susceptibility and suppressed the expression of secondary metabolite biosynthesis genes, while repressor lines showed enhanced expression of these genes [111]. Similarly, the ERF activator *CmHRE2-like* was aphid-inducible, and its overexpression increased susceptibility, while repressor lines enhanced resistance through modulation of flavonoid biosynthesis [112] (Table 3).

Several exogenous genetic resources and molecular approaches have been developed for improving insect resistance in chrysanthemum. Introduction of a modified *cry1Ab* gene conferred strong resistance to *H. armigera*, with high *cry1Ab*-accumulating lines causing complete larval mortality. Moreover, transgenic chrysanthemums carrying both modified *cry1Ab* and *sarcotoxin IA* genes exhibited strong resistance to lepidopteran larvae [113,114]. Introduction of the EBF synthase (*TcEbFS*) gene from pyrethrum enabled cultivated chrysanthemum to release the aphid alarm pheromone (E)-β-farnesene in susceptible tissues such as stems and young buds, thereby disrupting aphid feeding behavior and contributing to resistance [115]. Similarly, transfer of the *Pinellia ternata* agglutinin (PTA) gene enhanced aphid resistance, which was stably inherited in T1 progenies [116]. Overexpression of *TcCHS* from pyrethrum resulted in the production of volatile chrysanthemol and its glycoside derivative, both of which exhibited independent anti-aphid activity. These metabolites reduced *A. gossypii* probing and reproduction and strongly suppressed field populations, providing dual volatile and nonvolatile defenses [117]. In addition, RNA interference (RNAi) has emerged as an eco-friendly alternative to insecticides, although its efficiency depends on dsRNA design, target species, and environmental conditions. In *Nicotiana benthamiana*, chloroplast-expressed hpRNAs targeting *H. armigera* acetylcholinesterase accumulated intact dsRNAs and conferred strong resistance, while nuclear expression generated siRNAs with weaker protection, highlighting chloroplast engineering as a more effective TK-RNAi strategy [118,119] (Table 3).

### 5.3. Biocontrol Strategies Against Insect Pests in Chrysanthemum

Predatory mites are valuable biocontrol agents in chrysanthemum. *Neoseiulus cucumeris* provides partial suppression of western flower thrips and is most effective when combined with other measures in IPM programs. *Phytoseiulus persimilis* effectively controls two-spotted spider mites, and resistant populations can maintain or even improve reproductive performance, suggesting stable control potential [120,121]. The insidious flower bug *Orius insidiosus* is a key predator of western flower thrips on chrysanthemum, effectively controlling populations with small releases; its efficacy is unaffected by photoperiod and it can be integrated with spinosad for season-long use [122]. The earwig *Doru luteipes* also shows potential due to habitat overlap, and together with *O. insidiosus*, their complementary foraging times support combined use in thrips biocontrol [123]. The augmentative release of parasitoids such as *Encarsia formosa* is widely used for whitefly control in protected cropping systems. Climate chamber simulations based on future climate scenarios showed that *E. formosa* developed faster, parasitized more, but lived shorter under projected conditions, highlighting both potential and challenges for maintaining effective whitefly biocontrol in a changing climate [124]. Greenhouse studies highlight the potential of microbial and insect-based biocontrol strategies in chrysanthemum cultivation. *Trichoderma* sp. significantly improved plant growth, yield, and reduced pest damage, while *Beauveria bassiana* enhanced plant performance and lowered disease incidence, with combined application providing promising benefits for both productivity and resistance [125]. In parallel, the integration of *B. bassiana* with the soil-dwelling rove beetle *Dalotia coriaria* suppressed both foliar- and soil-dwelling stages of western flower thrips, suggesting particular value for early-season population management [126]. Laboratory assays indicated that several South African entomopathogenic nematodes, particularly *Steinernema yirgalemense* and *Heterorhabditis baujardi*, were highly virulent against soil-dwelling stages and could complete their life cycles in thrips, highlighting their potential as biocontrol agents [127]. These findings demonstrate the potential of integrating predators, entomopathogenic fungi, and nematodes as viable alternatives to chemical sprays.

Plant essential oils represent a promising approach for thrips management, acting either as allelochemicals that influence host selection or as botanical insecticides. Compounds with attractive, repellent, or deterrent activity can be sustainably applied through spraying or fumigation. For example, marjoram, clary sage, perilla, and spearmint oils exhibited insecticidal activity against *Thrips flavus*, with spearmint oil achieving complete control and attracting females. Their major constituents, including linalool, isopropyl myristate, limonene, and carvone, highlight Lamiaceae oils as promising candidates for thrips control [128,129]; Similarly, screening of nine essential oils identified 13 active compounds, primarily sesquiterpenes and monoterpenes, that were strongly associated with thrips mortality. Whole-plant assays confirmed that several of these oils significantly reduced thrips populations, with efficacy comparable to flonicamid, suggesting strong potential for greenhouse IPM applications [130]. In addition to thrips, botanical extracts have also shown efficacy against aphids in chrysanthemum. Leaf extracts of *Tithonia sinensis*, *T. diversifolia*, *Azadirachta indica*, and oil of *Cymbopogon nardus* effectively suppressed aphid populations, with *T. sinensis* extract providing the strongest suppression and enhancing flower opening, outperforming the synthetic insecticide imidacloprid [131]. Furthermore, extracts of *Chrysanthemum cinerariaefolium* demonstrated stable suppression of *A. gossypii* at higher concentrations, reinforcing their potential as eco-friendly options for aphid management in chrysanthemum [102].

## 6. Conclusions and Future Perspectives

Between 2015 and 2025, advances in molecular biology, genomics, and ecological regulation technologies have driven significant progress in the study and management of diseases and pests in chrysanthemum. Achievements span pathogen identification, elucidation of host resistance mechanisms, and the development of biocontrol strategies. In fungal diseases, molecular diagnostic approaches such as multi-locus DNA sequencing and multiplex real-time quantitative PCR have enabled precise classification of key pathogens, while resistance-regulating roles of certain transcription factor modules and hormone signaling pathways have been clarified. Biocontrol strategies, including *T. harzianum*, *B. subtilis*, and biochar combinations, have also demonstrated promising efficacy. Research on bacterial diseases has benefited from taxonomic revisions that clarified pathogen identities; although some technologies have reduced disease severity, the scarcity of strong genetic resistance resources remains a major constraint for breeding durable resistant cultivars. In the field of viral and viroid diseases, many novel viruses have been identified, while multiplex RT-PCR and RT-LAMP have enhanced detection efficiency. Virus-free plantlet propagation and RNAi-based transgenic techniques have been widely applied, yet broad-spectrum defense capacity is still limited. Regarding insect pests, the harmful effects of aphids and thrips, including their role in virus transmission, have been elucidated. Mechanisms of resistance regulated by epidermal trichome density, terpenoid metabolism, and specific transcription factors have been uncovered, while biocontrol agents such as predatory mites and *B. bassiana*, together with plant extracts, have been applied to pest suppression.

Future efforts in chrysanthemum disease and pest management should focus on integrated technologies and overcoming core bottlenecks. Priority directions include mining additional resistance gene resources and accelerating the breeding of durable resistant cultivars through the integration of molecular breeding and gene editing. Emerging technologies such as spray-induced gene silencing (SIGS) and chloroplast-mediated RNAi should be expanded to enhance defense against viruses and diverse insect pests. Ecological regulation and microbiome management require further development, including synergistic “biocontrol agent-biochar-plant” systems to suppress soilborne diseases, intercropping models with other crops, and the systematic evaluation of natural enemies and microbial agents with optimization of strain selection and release strategies. Finally, multi-omics analyses and predictive modeling can facilitate the construction of “resistance trait maps” to efficiently screen for highly resistant cultivars.

## Figures and Tables

**Table 1:** The Main Diseases and Pests of Chrysanthemum.

Fungal Diseases	Pathogen	Bacterial Diseases	Pathogen	Viral and Viroid Diseases	Viruses/Viroids	Insect Pests	Pest Species
Leaf spot	*Nigrospora oryzae, Nigrospora sphaerica*	Stem rot	*Dickeya chrysanthemi*	Viruses	CVB, CVR, CMV, CSNV, TSWV, INSV, PVY, TMV, TAV	Aphids	*Macrosiphoniella sanborni, Myzus persicae,* *Aphis gossypii*
Leaf blight	*Alternaria alternata*	Bacterial wilt	*Ralstonia solanacearum*	Viroids	CSVd, CChMVd	Thrips	*Frankliniella occidentalis*
Powdery mildew	*Golovinomyces cichoracearum*	Leaf spot/blight	*Pseudomonas cichorii*, *P. putida*			Whiteflies	*Trialeurodes vaporariorum, Bemisia tabaci*
Anthracnose	*Colletotrichum siamense*	Crown gall	*Agrobacterium rubi,* *A. tumefaciens*			Leafminers	*Liriomyza* spp. (*L. trifolii*, *L. huidobrensis*, *L. sativae*)
Gray mold	*Botrytis cinerea*	Leafy gall	*Rhodococcus fascians*			Spider mites	*Tetranychus urticae*
Wilt	*Fusarium incarnatum*					Lepidoptera pests	*Helicoverpa armigera*
White rust	*Puccinia horiana,* *P. chrysanthemi*						
Soil-borne diseases	*Sclerotinia sclerotiorum, Verticillium dahliae*						

**Table 3:** Physiological Defense and Resistance Research in Chrysanthemum against Insect Pests.

Category	Factor/Approach	Mechanism and Effect	References
Natural Resistance Traits	Leaf toughness, trichome density, secondary metabolites	Physical/chemical barriers; natural variation linked to higher resistance	[106]
	VOCs	Aphid-induced VOC shifts; species-specific preferences, compounds linked to resistance	[107]
Transcription Factors and Regulation	*CmWRKY48*	Aphid-inducible; overexpression suppressed aphid growth	[107]
	*CmMYB15*	Promotes lignin biosynthesis; overexpression enhanced resistance	[108,109]
	*CmMYB19*	Activates lignin pathway; restricted aphid multiplication	[108,110]
	*CmWRKY53*	Negative regulator; overexpression increased susceptibility, repression enhanced resistance	[111]
	*CmHRE2-like*	Regulates flavonoids; overexpression increased susceptibility, repression enhanced resistance	[111]
Exogenous Genes and Emerging Tech	*cry1Ab* + *sarcotoxin IA*	Produced insecticidal proteins; high-expression lines killed *H. armigera* and lepidopteran larvae	[113,114]
	*TcEbFS*	Synthesized aphid alarm pheromone (E)-β-farnesene; disrupted feeding	[115]
	*PTA* gene	Interfered with feeding; stable aphid resistance in transgenic lines	[116]
	*TcCHS*	Produced chrysanthemol and glycosides; suppressed *A. gossypii* probing and reproduction	[117]
	RNAi (chloroplast vs. nuclear)	Chloroplast dsRNA gave strong resistance; nuclear siRNA weaker	[118,119]

## Data Availability

No new data were created or analyzed in this study. Data sharing is not applicable to this article.
